# Development of 2-Methoxyhuprine as Novel Lead for Alzheimer’s Disease Therapy

**DOI:** 10.3390/molecules22081265

**Published:** 2017-07-28

**Authors:** Eva Mezeiova, Jan Korabecny, Vendula Sepsova, Martina Hrabinova, Petr Jost, Lubica Muckova, Tomas Kucera, Rafael Dolezal, Jan Misik, Katarina Spilovska, Ngoc Lam Pham, Lucia Pokrievkova, Jaroslav Roh, Daniel Jun, Ondrej Soukup, Daniel Kaping, Kamil Kuca

**Affiliations:** 1 Biomedical Research Centre, University Hospital Hradec Kralove, Sokolska 581, 500 05 Hradec Kralove, Czech Republic; eva.mezeiova@gmail.com (E.M.); korabecny.jan@gmail.com (J.K.); vsepsova@gmail.com (V.S.); martina.hrabinova@unob.cz (M.H.); petr.jost@unob.cz (P.J.); rafael.dolezal@fnhk.cz (R.D.); honzamisik@seznam.cz (J.M.); k.spilovska@gmail.com (K.S.); phama92@gmail.com (N.L.P.); daniel.jun@unob.cz (D.J.); ondrej.soukup@fnhk.cz (O.S.); 2 National Institute of Mental Health, Topolova 748, 250 67 Klecany, Czech Republic; Daniel.Kaping@nudz.cz; 3 Department of Toxicology and Military Pharmacy, Faculty of Military Health Sciences, University of Defence in Brno, Trebesska 1575, 500 01 Hradec Kralove, Czech Republic; lubica.muckova@unob.cz (L.M.); kucera-t@email.cz (T.K.); 4 Department of Organic and Biorganic Chemistry, Faculty of Pharmacy in Hradec Kralove, Charles University, Heyrovskeho 1203, 500 05 Hradec Kralove, Czech Republic; elpokrievkova@gmail.com (L.P.); rohj@faf.cuni.cz (J.R.); 5 Department of Chemistry, Faculty of Science, University of Hradec Kralove, Rokitanskeho 62, 500 03 Hradec Kralove, Czech Republic

**Keywords:** acetylcholinesterase, Alzheimer’s disease, butyrylcholinesterase, tacrine, 7-MEOTA, huprine Y, 2-methoxyhuprine

## Abstract

Tacrine (THA), the first clinically effective acetylcholinesterase (AChE) inhibitor and the first approved drug for the treatment of Alzheimer’s disease (AD), was withdrawn from the market due to its side effects, particularly its hepatotoxicity. Nowadays, THA serves as a valuable scaffold for the design of novel agents potentially applicable for AD treatment. One such compound, namely 7-methoxytacrine (7-MEOTA), exhibits an intriguing profile, having suppressed hepatotoxicity and concomitantly retaining AChE inhibition properties. Another interesting class of AChE inhibitors represents Huprines, designed by merging two fragments of the known AChE inhibitors—THA and (−)-huperzine A. Several members of this compound family are more potent human AChE inhibitors than the parent compounds. The most promising are so-called huprines X and Y. Here, we report the design, synthesis, biological evaluation, and in silico studies of 2-methoxyhuprine that amalgamates structural features of 7-MEOTA and huprine Y in one molecule.

## 1. Introduction

Alzheimer’s disease (AD) is neurodegenerative disorder which manifests itself in neurocognitive dysfunction (e.g., memory loss, behavioral abnormalities, depression, hallucinations, delusion, and agitation). The neuropathological changes associated with AD ultimately lead to consistent irreversible loss of neurons and synapses throughout the brain. This consequently results in the aggravation of AD symptoms and finally in patient’s death [1]. AD is considered one of the major public health issues due to the lack of effective treatment and demographic aging, which will increase the number of people suffering from AD in the future. The economic impact of AD is another important fact to consider [2,3].

**Figure 1:**
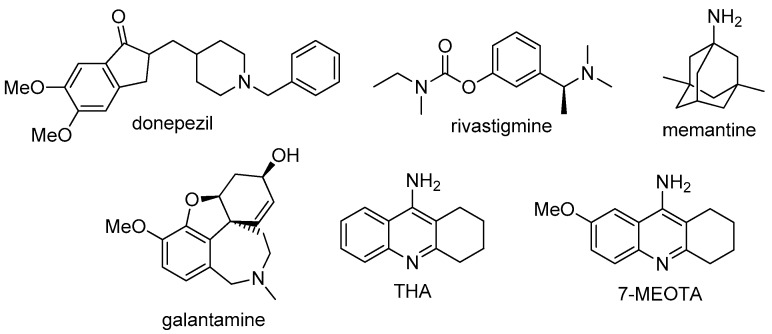
Structures of currently available drugs for the treatment of AD—donepezil, rivastigmine, memantine, and galantamine, and structures of THA and its methoxy derivative 7-MEOTA.

The development of effective AD drug treatment builds upon pathomechanism understanding. One of the oldest hypothesis describing mechanisms of AD development support cholinergic dysfunction [1]. Accordingly, the alterations in cholinergic system (such as reduction of activity of different enzymes that are responsible for the synthesis end release of neurotransmitter acetylcholine (ACh)) lead to degeneration of cholinergic neurons and the disruption of cholinergic neurotransmission. Currently approved compounds for the treatment of mild to moderate AD stages—donepezil, rivastigmine, and galantamine provide symptomatic treatment by improving cholinergic neurotransmission via inhibition of acetylcholinesterase (AChE, E.C. 3.1.1.7.) to improve the quality of life of AD patients [1,4] (Figure 1).

Besides AChE inhibitors (AChEIs), memantine (Figure 1) has been approved for moderate to severe stages of AD treatment. Memantine acts as an *N*-methyl-d-aspartate receptor (NMDAr) antagonist protecting against glutamate mediated excitotoxicity [1,4,5,6]. Also other approaches are currently extensively pursued; among others inhibitors of beta-secretase [7,8,9], direct inhibition of amyloid beta (Aβ) overproduction [10], or their combination into one entity [11,12].

Other neuropathological changes associated with AD are intracellular neurofibrillary lesions composed of hyperphosphorylated tau protein and extracellular accumulation of Aβ plagues [1,13,14,15]. Despite research efforts to develop novel therapeutics addressing Aβ overload or tau hyperphosphorylation, it still lacks effectiveness resulting in AChEIs and memantin to remain the only approved AD drugs [16].

The first clinically used drug for the treatment of AD, THA (1,2,3,4-tetrahydro-9-aminoacridine), is centrally active, reversible AChEI. It was withdrawn from the market due to its poor oral bioavailability, the necessity of multiple day-doses, and a number of serious side effects such as hepatotoxicity, nausea, diarrhea, urinary incontinence, and potential carcinogenicity [17]. However, THA remains interesting for medicinal chemists involved in AD research as a scaffold for the development of novel lead compounds with better toxicological profile than the parent molecule. With this in mind, a THA derivative, 7-MEOTA, was developed [18,19]. 7-MEOTA retained the pharmacological profile of THA with lower occurrence of side effects. This lower toxicity can be ascribed to having a different metabolic pathway [17,20].

## 2. Design

**Figure 2:**
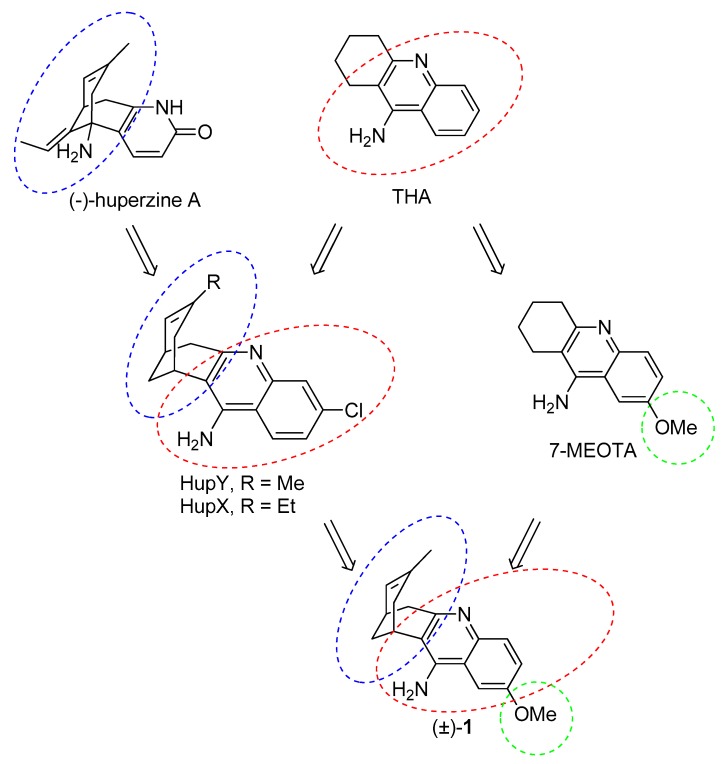
Design strategy towards 2-methoxyhuprine (±)-**1**.

The essential idea behind the design of huprines was to find a lead molecule that will be able to span the active side of AChE as much as possible [21]. To achieve this aim, two known AChEIs were chosen. Indeed, huprines amalgamate structural motifs of THA (4-aminoquinoline system) and (−)-huperzine A (carbobicyclic bridged moiety). The development of huprines has been conceived empirically without any clear idea about the occupancy of huprine in the AChE active site. Further research has uncovered a divergent binding site for huprines as was predicted in the initial hypothesis. This fact has been corroborated in the following years by resolving 3D structures of the *Torpedo californica* AChE (*Tc*AChE) enzyme with THA [22], (−)-huperzine A [23] and (−)-huprine X [24]. In this regard, huprine X (HupX) and Y (HupY) (Figure 2) represent the first generation of huprine analogues. These compounds are more than 100 times more potent AChEIs than the parent structures [21]. HupY ameliorated the signs of intoxication mediated via 3-nitropropionic acid (3-NP), natural toxin inducing neurodegeneration [25]. More importantly, it increased the survival of HupY pretreated mice in a 3-NP-induced assay without any alteration in body weight or muscular activity [26]. All these findings point to a neuroprotective action of HupY. Taking all these advantages together, we have combined the structural features of HupY with 7-MEOTA by exploiting a merging approach to yield 2-methoxyhuprine ((±)-**1**) (Figure 2). In this article, we report on the synthesis, biochemical studies, including cholinesterase inhibition, preliminary toxicity on HepG2 cell line, and in silico study in the human AChE (*h*AChE) and human BChE (*h*BChE) active sites.

## 3. Results and Discussion

### 3.1. Synthesis

**Scheme 1:**
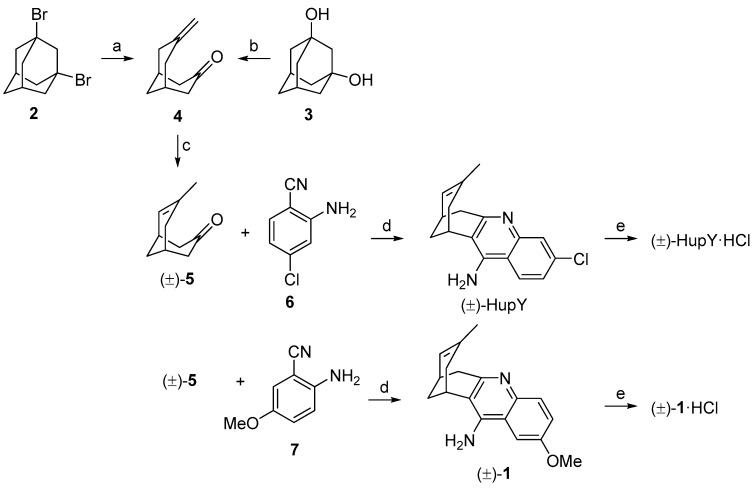
Synthesis of (±)-HupY·HCl and (±)-**1**·HCl. Reagents and conditions: (a) 1M NaOH/1,4-dioxane (1:1), 170 °C, 18 h, 82%; (b) *p*-TsCl, DMAP, toluene, 130 °C (MW), 1.5 h, 72%; (c) 5% Pd/C, H_2_, EtOH, r.t., 2 h; (d) **6** or **7**, AlCl_3_, 1,2-dichloroethane, (±)-HupY, 81% after two steps, (±)-**1**, 80% after two steps; (e) HCl, methanol, rt, overnight, (±)-HupY·HCl, 91%, (±)-**1**·HCl, 94%.

The synthetic approach for the preparation of (±)-HupY and its 2-methoxy derivative (±)-**1** is depicted in Scheme 1. The synthesis followed the known two-step protocol [27,28]. The fragmentation reaction of commercially available 1,3-dibromoadamantane (**2**) or 1,3-adamantanediol (**3**) generated ketone **4** in 82% and 77% yields, respectively. Ketone **4** containing a vinyl functional group was than treated with 5% Pd/C in ethanol under hydrogen atmosphere. The isomerization of double bond under this condition led to a racemic mixture of compound (±)-**5** [28]. The crude product was used without any further purification in the next reaction. The final products were obtained by Friedlander-type condensation of (±)-**5** with 2-amino-4-benzonitrile (**6**) or 2-amino-5-methoxybenzonitrile (**7**) in the presence of aluminium trichloride as catalyst in 1,2-dichloroethane to yield (±)-HupY or (±)-**1**, respectively [29]. Both huprine derivatives (±)-HupY and (±)-**1** were then converted into corresponding hydrochlorides.

### 3.2. Evaluation of AChE and BChE Inhibition Activity

AChE and BChE are members of the serine hydrolase family. They are responsible for the breakdown of choline-based structures being implicated in neurotransmission like ACh degradation by AChE/BChE or in detoxification of xenobiotics like cocaine, salicylic acid, and others by BChE [30,31]. Imbalance in ACh neurotransmission is regarded as one contributing aspect to the manifestation of symptoms related to disorders like AD, myasthenia gravis, glaucoma, Parkinson’s disease, etc. [32,33]. Accordingly, ChEIs received particular attention in attenuating the symptoms in neurodegenerative disorders not only in AD [4].

The inhibitory activity of (±)-**1**·HCl towards *h*AChE and *h*BChE was determined by the modified spectrophotometric method described by Ellman et al. [34,35,36,37]. THA, 7-MEOTA, 6-chlorotacrine, and (±)-HupY·HCl were used as reference compounds. The results are summarized in Table 1 and are expressed as IC_50_ values.

Initially, obtained results for the reference compounds are in good agreement with the literature data [19,38,39]. 2-Methoxyhuprine (±)-**1**·HCl showed inhibitory activity on a micromolar scale towards both ChEs. (±)-**1**·HCl displayed three orders of magnitude less potent inhibition than (±)-HupY·HCl against *h*AChE which, in turn, revealed inhibitory activity on a one-digit nanomolar scale. 6-Chlorotacrine exerted two-digit nanomolar inhibition power, being two-fold more potent than (±)-**1**·HCl. (±)-**1**·HCl yielded one order higher activity than 7-MEOTA, however, still being slightly less active than THA.

The present knowledge of pathophysiological mechanisms behind AD permits potential design of AChE-selective, non-selective ChE, and BChE-selective inhibitors [40]. The question, which remains open, is whether to focus our efforts on developing novel agents of a certain family of ChEIs. Selective BChEIs may have higher benefits in patients with moderate-to-severe stages of AD when the levels of AChE are depleted and BChE substitutes its role [41]. BChEIs can also mitigate the burden of Aβ [42]. In the case of AChE, the similar mode of action (Aβ inhibition assembly) provided by direct AChE inhibition through its peripheral anionic site (PAS) gave rise to the so-called dual binding site strategy, i.e., ligands capable to interact with both anionic sites concomitantly [43,44]. Significantly, (±)-**1**·HCl demonstrated one-order higher *h*BChE affinity than 7-MEOTA and comparable potency to that of 6-chlorotacrine. (±)-**1**·HCl falls one order of magnitude behind (±)-HupY as well as THA, the latter having the highest inhibitory potency towards *h*BChE among all the tested compounds. Non-selective profile of (±)-**1**·HCl further underline the significance of this compound to be potentially used in any stages of AD.

### 3.3. Kinetic Study of AChE Inhibition

To evaluate the interactions of novel derivative (±)-**1**·HCl with *h*AChE, a kinetic study was performed. Enzyme velocity curves were measured at various concentrations of substrate and tested compound. The type of inhibition was elucidated from the nonlinear regression analysis. Results for each type model of inhibition (competitive, non-competitive, uncompetitive, and mixed) were compared with sum-of-squares *F*-test.

**Figure 3:**
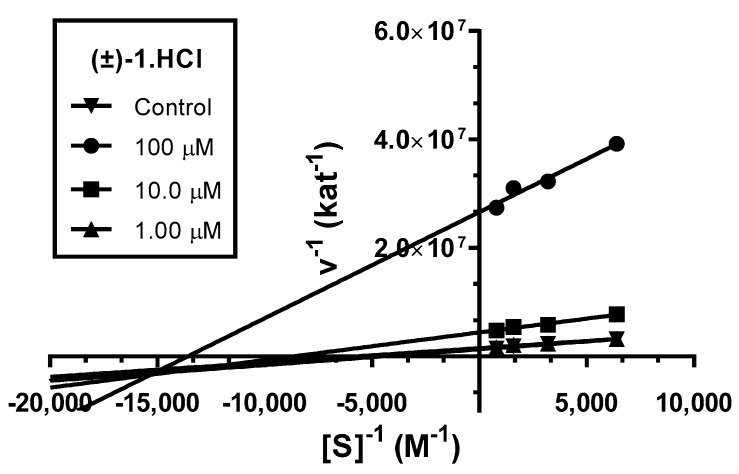
Steady-state inhibition of *h*AChE hydrolysis of substrate acetylthiocholine by novel huprine derivative (±)-**1**·HCl at different concentrations. Lineweaver−Burk plots of initial velocity at increasing substrate concentrations (0.156–1.250 mM) are presented. Lines were derived from a linear regression of the data points.

Analysis indicated mixed type of inhibition (*p* ˂ 0.05), consistent with graphical representation of Lineweaver–Burk plot in the Figure 3. The same type of inhibition was described in literature for parent compound HupY [49].

The intersection of lines is placed below the x-axis. This indicates higher affinity to the enzyme–substrate complex than to the free enzyme (*K*_i_ > *K*_i’_). *K*_m_ was slightly decreased, while the value of *V*_max_ decreased with higher concentration of (±)-**1**·HCl. This means that the inhibitor binds reversibly to both the free enzyme and enzyme–substrate complex, affecting the binding of the substrate in the active site of *h*AChE and interacting with PAS. This causes conformational changes of the enzyme resulting also in changes in the active site. A corresponding *K*_i_ value of 16.4 ± 1.3 µM and *K*_i’_ of 3.67 ± 0.42 µM was measured.

### 3.4. In Vitro Blood-Brain Barrier Permeation and Druglikeness

Anti-Alzheimer drugs are postulated to be centrally active, thus their effective permeation is necessary. To investigate the possibility of new huprine derivative (±)-**1**·HCl to cross the blood-brain barrier (BBB), a parallel artificial membrane permeation assay (PAMPA) was used [50,51]. (±)-HupY is able to cross the BBB as suggested by previous ex vivo [39,49] and in vivo studies [26]. Our results from Table 2 clearly demonstrate that (±)-**1**·HCl as well as (±)-HupY·HCl should be able to cross the BBB by passive diffusion, having *P*_e_ values above the standard drugs which are known to be CNS available (THA and donepezil). Negative controls (cefuroxime and piroxicam) are also reported. The higher *P*_e_ of (±)-**1**·HCl is indicative of its higher lipophilicity caused by the introduction of methoxy group.

In order to address drug likeness of novel huprine (±)-**1**·HCl, we have calculated its suitability in terms of balanced physicochemical properties, applying the CNS multiparameter optimization (MPO) algorithm (Table 3) [54]. This smart tool has been validated on a large dataset mainly composed from the library of Pfizer CNS drug candidates as well as marketed drugs. CNS MPO score uses a set of six physicochemical parameters (i) lipophilicity expressed as partition coefficient (Clog*P*); (ii) calculated distribution coefficient at pH = 7.4 (Clog*D*_7.4_); (iii) molecular weight (MW); (iv) topological polar surface area (TPSA); (v) number of hydrogen bond donors (HBD); and (vi) p*K*_a_ value for the most basic center in the molecule (Cp*K*_a_) [55]. All these parameters have been calculated by MarvinSketch 15.1.19.0 (Budapest, Hungary). The versatility of this tool lies in expanding the space for CNS active compounds where the violation of one of these psychicochemical characteristics does not necessarily mean the complete compounds exclusion from further testing. Following the CNS MPO algorithm, the calculated values for each specific psychicochemical parameter as well as CNS desirability can be found in Table 3. Accordingly, CNS MPO endpoints indicated that all the reference compounds as well as (±)-**1**·HCl fulfill the criteria for CNS drug candidates (CNS MPO = 4–6).

### 3.5. Cytotoxicity

To evaluate the cytotoxic profile of 2-methoxyhuprine (±)-**1**·HCl and other AChEIs under the survey, a 3-(4,5-dimethylthiazol-2-yl)-2,5-diphenyltetrazolium bromide (MTT) assay [56] was carried out on human liver carcinoma (HepG2), human renal adenocarcinoma (ACHN), and human neuroblastoma (SH-SY5Y) cell lines. The data obtained from these measurements are summarized in Table 4. THA has been shown to cause reversible abnormalities in liver function, and the exact mechanism of its action toward hepatocytes has not been fully elucidated. However, the relationship between metabolic activation to its protein-reactive form and cytotoxic metabolites has been found in a study performed with human liver microsomes [57,58]. Some studies pointed out that THA induces early alteration in membrane fluidity, a process related to cytotoxicity [59]. The disrupted membrane integrity results in lactate dehydrogenase (LDH) leakage while the occurrence of alanine aminotransferase (ALT) elevation was reported as late-onset [60]. Keeping this in mind, we have evaluated the potential cytotoxic effect of novel huprine (±)-**1**·HCl and all the reference compounds. We found out that THA was the least toxic compound with IC_50_ values among 120–168 µM for all the tested cell lines. The therapeutic plasma concentration of THA (7–20 ng/mL) associated with elevation in liver aminotransferases in human is substantially lower than in vitro cytotoxicity concentrations [58,61]. The second ranked compound with the lowest cytotoxicity can be considered 7-MEOTA and 6-chlorotacrine. Focusing on (±)-**1**·HCl, the data indicated the similar cytotoxic profile as that found for (±)-HupY·HCl on HepG2 cell line, however, (±)-HupY·HCl is more cytotoxic than 7-MEOTA. Note that 7-MEOTA is considered a relatively safe THA derivative as proved in many in vivo studies [17,19,20,45,62]. Other important findings related to ACHN and SH-SY5Y cell lines suggested that (±)-**1**·HCl cytotoxicity lies behind the cytotoxicity profile of 6-chlorotacrine. This two kidney- and neuronal-origin cells lines were found more sensitive to both (±)-**1**·HCl and (±)-HupY·HCl, respectively. From this point of view, molecular weight increment goes hand in hand with increasing cytotoxicity against all three cell lines. Taken together, this preliminary data has to be taken with precaution since they could not reflect the real toxicity profile of the compound under in vivo conditions.

### 3.6. In Silico Docking Studies

The aforementioned biological data for (±)-**1**·HCl and (±)-HupY·HCl represent the sum of effects for both enantiomers. Since the separation of the racemic mixture into pure enantiomers and successive biological evaluation was not feasible in our hands, we decided to inspect the binding modes for each enantiomer of **1**·HCl and compared it with HupY enantiomers. From the literature sources available, we found out that the more active isomer was (−)-HupY (correspond to 7*S*,11*S*-HupY), possessing *h*AChE IC_50_ = 0.32 nM, over (+)-HupY (7*R*,11*R*-HupY) with *h*AChE IC_50_ = 123 nM. The same observation can be made for *h*BChE where (+)-HupY was slightly more active than (−)-HupY ((+)-HupY *h*BChE IC_50_ = 153 nM; (−)-HupY *h*BChE IC_50_ = 247 nM) [39]. In order to predict more potent *h*AChE and *h*BChE enantiomer of **1**·HCl we have carried out the molecular modeling simulation on the models of *h*AChE (PDB ID: 4EY7) and *h*BChE (PDB ID: 4BDS) into their active sites [63,64].

**Figure 4:**
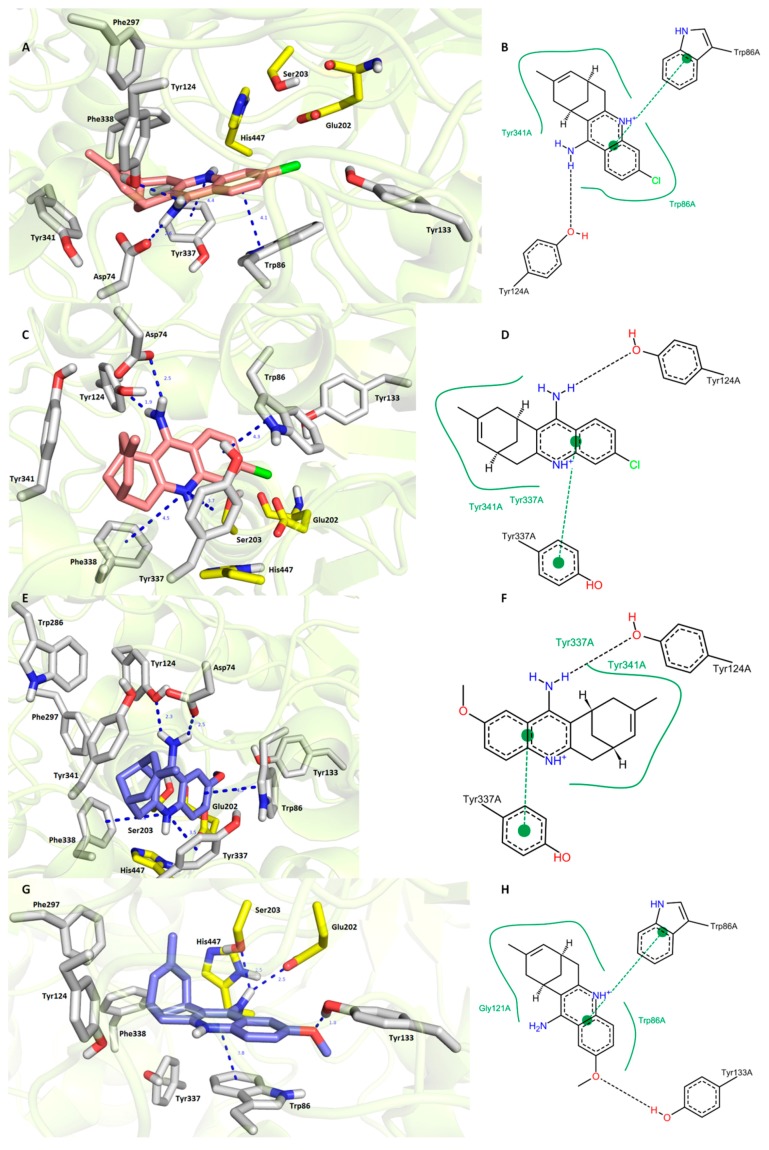
Docking results for HupY ((**A**,**B**)—7*R*,11*R*-HupY; (**C**,**D**)—7*S*,11*S*-HupY) and **1** ((**E**,**F**)—7*S*,11*S*-**1**; (**G**,**H**)—7*R*,11*R*-**1**) within *h*AChE active site (PDB ID: 4EY7). Generally to (**A**,**C**,**E**,**G**)—HupY is rendered in salmon carbon atoms; **1** is depicted in dark blue carbon atoms; important amino acid residues involved in the ligand–enzyme interactions are displayed in grey carbon atoms; catalytic triad residues (Glu202, Ser203, His447) are shown in yellow; rest of the enzyme is represented in grey cartoon; (**B**,**D**,**F**,**H**)—2D representation of binding modes of HupY and **1**. (**B**,**D**,**F**,**H**) were created with PoseView software (PoseView, http://poseview.zbh.uni-hamburg.de/poseview/wizard, 2012); (**A**,**C**,**E**,**G**) were generated with PyMol 1.30 (The PyMOL Molecular Graphics System, Version 1.5.0.4 Schrödinger, LLC.).

Paying attention to *h*AChE (Figure 4), it has to be noted that 7*S*,11*S*-HupY as well as 7*R*,11*R*-HupY shared a high degree of spatial conformity. This includes (i) parallel π-π stacking against Trp86; (ii) hydrogen bonding between an exocyclic amino group from HupY and an amino group from Asp74, as well as a hydroxyl group from Tyr124; and (iii) hydrophobic interaction with Tyr341. 7*S*,11*S*-HupY (Figure 4C,D) exerted slightly more favorable orientation than 7*R*,11*R*-HupY (Figure 4A,B) in the *h*AChE active site. This presumably stems from a closer spatial arrangement of HupY aromatic core to Tyr337 via π-π/cation-π contact (3.7 Å for 7*S*,11*S*-HupY; 4.4 Å for 7*R*,11*R*-HupY) being attributed to less steric hindrance of carbobicyclic huprine moiety. Moreover, 7*S*,11*S*-HupY adapted parallel cation-π contact with Phe338. This type of interaction was not provided by 7*R*,11*R*-HupY. Estimated binding energies for each ligand were computed by Autodock Vina as follows −12.6 kcal/mol and −12.3 kcal/mol for 7*S*,11*S*-HupY and 7*R*,11*R*-HupY, respectively. Our results can be put into contrast with huprine derivative, namely HupX bearing ethyl appendage instead of methyl in HupY [24]. In this case, (−)HupX was co-crystalized with *Tc*AChE indicating sandwich π-π stacking between Trp84 and Phe330. This type of interaction is analogous to the lodging of 7*S*,11*S*-HupY (see Phe338 and Trp86, Figure 4C). In HupX–*Tc*AChE complex, conserved water molecules mediated hydrogen bonding between amino group and some amino acid residues. Since we did not include water molecules into our experiments, the latter cannot be confirmed for the HupY–*h*AChE complex. Lastly, methyl, carbobicyclic scaffold, and chlorine from (−)-HupY (Figure 4C,D) filled the same regions as HupX in *Tc*AChE. All these findings underlie the validity of our experimental protocol.

Completely different poses were obtained for 7*S*,11*S*-**1** (Figure 4E,F) and 7*R*,11*R*-**1** (Figure 4G,H) in *h*AChE active site. Better ligand fitting was found for 7*S*,11*S*-**1** (−12.3 kcal/mol) over 7*R*,11*R*-**1** (−11.6 kcal/mol). Interestingly, anchoring of 7*S*,11*S*-**1** resembles the accommodation of 7*S*,11*S*-HupY in *h*AChE CAS subsite with several specific features outlined previously. Compared to 7*S*,11*S*-**1**, typical hydrogen bonds between exocyclic amino group of **1** and Asp74 and Tyr124 diminished when 7*R*,11*R*-**1** was embedded into the *h*AChE active site. On the contrary, 7*R*,11*R*-**1** was engaged in hydrogen bond formation between the methoxy group of 7*R*,11*R*-**1** and the hydroxyl of Tyr133 (1.8 Å) together with connection of the amino group of 7*R*,11*R*-**1** to Ser203 (2.5 Å) and Glu202 (2.5 Å), amino acid residues from the catalytic triad. Taking all these findings and aspects together and putting them in contrast with the known activity facts for each enantiomer of HupY, we assume that the more active *h*AChE enantiomer of **1** relates to 7*S*,11*S*-**1**.

**Figure 5:**
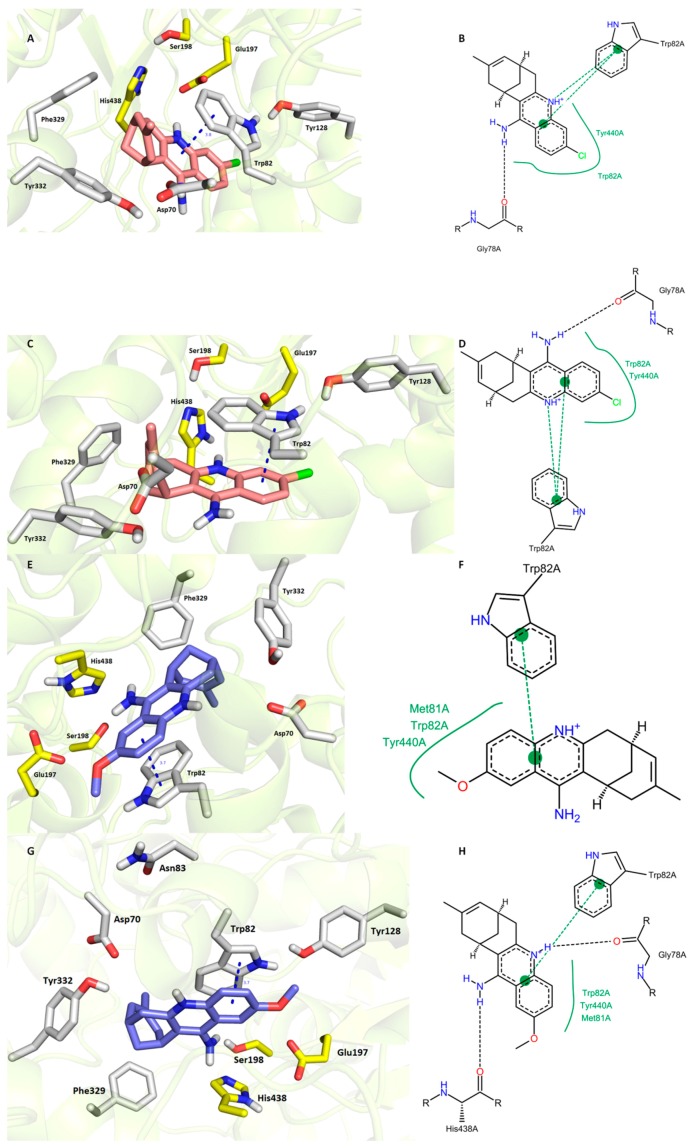
Docking results for HupY ((**A**,**B**)—7*R*,11*R*-HupY; (**C**,**D**)—7*S*,11*S*-HupY) and **1** ((**E**,**F**)-7*S*,11*S*-**1**; (**G**,**H**)—7*R*,11*R*-**1**) within *h*BChE active site (PDB ID: 4BDS). Generally to A, C, E, G—HupY is rendered in salmon carbon atoms; **1** is depicted in dark blue carbon atoms; important amino acid residues involved in the ligand-enzyme interactions are displayed in grey carbon atoms; catalytic triad residues (Glu198, Ser197, His438) are shown in yellow; rest of the enzyme is represented in grey cartoon; (**B**,**D**,**F,**,**H**)—2D representation of binding modes of HupY and **1**. (**B**,**D**,**F,**,**H**) were created with PoseView software (PoseView, http://poseview.zbh.uni-hamburg.de/poseview/wizard, 2012); (**A**,**C**,**E**,**G**) were generated with PyMol 1.30 (The PyMOL Molecular Graphics System, Version 1.5.0.4 Schrödinger, LLC.).

Very close IC_50_ values were experimentally determined for 7*S*,11*S*-HupY and 7*R*,*11*R-HupY against *h*BChE [39]. Our docking procedure was not able to sufficiently explain discrepancies behind the orientation of each enantiomer in *h*BChE active site (7*R*,*11*R–HupY–*h*BChE complex is superimposed in Figure 5A,B; 7*S*,*11*S–HupY–*h*BChE complex is rendered in Figure 5C,D). In general, the common structural features can be outlined as follows (i) face-to-face orientation of **1** aromatic part against Trp82 (3.8 Å and 3.6 Å for 7*R*,*11*R-HupY and 7*S*,*11*S-HupY, respectively); (ii) hydrogen bond contact with carbonyl group from Gly78; and (iii) hydrophobic contact Trp82 and Tyr440. According to the calculated binding affinities, the more preferred *h*BChE binder can be highlighted as 7*R*,*11*R-HupY over 7*S*,*11*S-HupY with estimated binding energies −11.1 kcal/mol and −10.7 kcal/mol, respectively. This is also in line with data from literature [39].

The docking simulation for **1** was able to distinguish and predict the more active enantiomer against *h*BChE (Figure 5E,F 7*S*,11*S*-**1**; G,H 7R,11*R*-**1**). Although both enantiomers occupied the same region of *h*BChE, making π-π contact with Trp82 in the same distance 3.7 Å, few more interactions between ligand and enzyme can be observed for 7R,11*R*-**1**. These are hydrogen bond connections between the heterocyclic nitrogen of 7R,11*R*-**1** with the carbonyl group from Gly78 and the exocyclic amino group of 7R,11*R*-**1** and His438 from the catalytic triad. In the case of 7*S*,11*S*-**1**, the catalytic triad remained unaffected. The latter statement is also supported by the calculated binding energies for 7R,11*R*-**1** and 7*S*,11*S*-**1**, which were −11.3 kcal/mol and −10.5 kcal/mol, respectively. However, the real potential of each enantiomer has to be determined by in vitro experiments to unequivocally assess their inhibition capacity.

## 4. Materials and Methods

### 4.1. General Chemistry Methods

All chemical reagents and solvents were used in the highest available purity without further purification and were purchased from Sigma-Aldrich (Prague, Czech Republic). The reactions were monitored by thin layer chromatography (TLC) on silica gel plates (60 F254, Merck, Prague, Czech Republic) and the spots were visualized by either spraying with a solution of *p*-anisaldehyde and subsequent heating or with ultraviolet light (254 nm). A CEM Discover SP focused microwave reactor was used for microwave-mediated reactions. Purifications of crude products were carried out using silica gel columns (silica gel 100, 0.063–0.200 mm, 70–230 mesh ASTM, Fluka, Prague, Czech Republic). The microwave-mediated reactions were performed using a CEM Discover SP focused microwave reactor. NMR spectra were recorded in deuterated chloroform (CDCl_3_), deuterated methanol (CD_3_OD) or deuterated dimethyl sulfoxide (DMSO-*d*_6_) on a Varian S500 spectrometer. Chemical shifts (δ) are reported in parts per millions (ppm) and spin multiplicities are given as singlet (s), broad singlet (bs), doublet (d), or multiplet (m). Coupling constants (*J*) are reported in Hz. Melting points were determined using automated melting point recorder M-565 (Büchi, Switzerland) and are uncorrected. The synthesized compound (±)-**1**·HCl was analyzed by LC-MS system consisting of UHPLC Dionex Ultimate 3000 coupled with Q Exaxtive Plus mass spectrometer to obtain high resolution mass spectra (Thermo Fisher Scientific, Bremen, Germany). Elemental analyses were obtained in a satisfactory range, confirming >95% purity.

#### 4.1.1. Preparation of 7-Methylenebicyclo[3.3.1]nonan-1-one (**4**)

A suspension of **2** (200 mg, 0.68 mmol) in 4.2 mL of a mixture of 1M NaOH/1,4-dioxane (1:1) was stirred at 175 °C for 18 h in an autoclave. The mixture was poured into ethyl-acetate (8 mL) and washed with water (4 mL). The aqueous phase was washed with another portion of ethyl-acetate (8 mL). The combined organic layers were dried over anhydrous Na_2_SO_4_ and concentrated under reduced pressure. Crude product was purified by column chromatography (petroleum-ether/ethyl-acetate, 4:1). Ketone **4** (84 mg) was obtained in 82% as white crystals.

Alternative approach for **4**: A suspension of **3** (250 mg, 1.49 mmol), *p*-TsCl (708 mg, 3.74 mmol) and DMAP (454 mg, 3.74 mmol) in dry toluene (5.4 mL) was stirred at 130 °C for 1.5 h under MW conditions. The crude reaction mixture was filtered-off and filtrate was concentrated under reduced pressure. The ketone was purified by column chromatography (petroleum-ether/ethyl-acetate, 4:1) to afford compound **4** (172 mg) in 77% yield. 

^1^H- and ^13^C-NMR spectra were in good agreement with published data [27,28].

#### 4.1.2. Preparation of *rac*-7-Chloro-15-methyl-10-azatetracyclo[11.3.1.0^2,11^.0^4,9^]heptadeca-2(11),3,5,7,9,14-hexaen-3-amine hydrochloride ((±)-HupY·HCl)

A suspension of ketone **4** (105 mg, 0.70 mmol) and 5% Pd/C (19.4 mg) in absolute ethanol (2.1 mL) was stirred under H_2_ atmosphere for 2 h. After the end of isomerization, the catalyst was filtered-off using filter paper and the filtrate was concentrated under reduced pressure. The crude product (±)-**5** was used to the following reaction without any purification. ^1^H and ^13^C-NMR spectra were in good agreement with published data [27,28].

To the solution of compound (±)-**5** (92 mg, 0.615 mmol) in 1,2-dichloroethane (1 mL), anhydrous AlCl_3_ (123 mg, 0.92 mmol) and **6** (117 mg, 0.77 mmol) were added. The reaction mixture was stirred at 95 °C (MW—50 W, 300 psi, high stirring) for 2 h. After cooling down to room temperature, THF (5 mL) and water (2.5 mL) were added, followed by addition of 2M aq. solution of NaOH to adjust pH to 8–9. The resulting mixture was stirred for additional 30 min. The aq. phase was washed with dichloromethane (2 × 8 mL). Combined organic layers were dried over anhydrous Na_2_SO_4_ and concentrated under reduced pressure. The residue was submitted to column chromatography (petroleum-ether/ethyl-acetate, 4:1) to give (±)-HupY (161 mg, 81% yield in two steps) as a slightly yellow solid.

Compound (±)-HupY (70 mg) was dissolved in methanol (1 mL) and then 2 M HCl in methanol (1 mL) was added. The mixture was stirred over night at room temperature. The solvents were evaporated under reduced pressure. The final product (±)-HupY·HCl (72 mg) was obtained in 91% yield. Spectral data were in good agreement with published data [29].

#### 4.1.3. Preparation of *rac*-6-Methoxy-15-methyl-10-azatetracyclo[11.3.1.0^2,11^.0^4,9^]heptadeca-2(11),3,5,7,9,14-hexaen-3-amine hydrochloride ((±)-**1**·HCl)

To the solution of ketone **4** (108 mg, 0.72 mmol) in absolute ethanol (2.2 mL) 5% Pd/C (20 mg) was added. The reaction mixture was stirred under H_2_ atmosphere for 2 h. The catalyst was removed by filtration through filter paper and the filtrate was concentrated under reduced pressure. Compound (±)-**5** was used to the next reaction without further purification. ^1^H- and ^13^C-NMR spectra were in good agreement with published data [27,28].

Ketone (±)-**5** (95 mg, 0.63 mmol) was dissolved in dry 1,2-dichloroethane (1 mL). Anhydrous AlCl_3_ (126.5 mg, 0.95 mmol) and **7** (121 mg, 0.79 mmol) were added to this solution. The mixture was stirred under MW (50 W, 300 psi, high stirring) conditions at 95 °C for 2 h. After the completion of the condensation, mixture of THF/H_2_O (7.5 mL, 2:1) was added, followed by 2 M aq. solution of NaOH adjusting pH to 8–9. The resulting mixture was stirred for 30 min. The organic and aqueous phases were separated and the aqueous one was washed with dichloromethane (2 × 8 mL). Combined organic layers were dried over anhydrous Na_2_SO_4_ and the solvent was removed under reduced pressure. The crude product was purified by column chromatography (CH_2_Cl_2_/MeOH/TEA, 95:5:1). Compound (±)-**1** (142 mg) was obtained in 80% yield in two steps as a white solid.

2 M HCl in methanol (1 mL) was added to the solution of (±)-**1** (70 mg, 0.25 mmol) in methanol (1 mL). The reaction mixture was stirred overnight and then solution was evaporated under reduced pressure. Hydrochloride (±)-**1**·HCl (74 mg) was obtained in 94% yield as a slightly yellow solid, m.p. 280 °C compound decomposition. ^1^H-NMR (DMSO-*d*_6_): δ 7.97 (d, *J* = 2.7 Hz, 1H, H_1_), 7.90 (d, *J* = 9.2 Hz, 1H, H_4_), 7.48 (dd, *J* = 9.2, 2.6 Hz, 1H, H_3_), 5.49 (d, *J* = 5.2 Hz, 1H, H_8_), 3.91 (s, 3H, OCH_3_), 3.35 (bs, 1H, H_11_), 3.13 (dd, *J* = 17.8, 5.5 Hz, 1H, H_6_), 2.91 (m, 1H, H_6_), 2.68 (bs, 1H, H_7_), 2.38 (m, 1H, H_10_), 1.85 (m, 3H, H_10_, 2 × H_13_), 1.50 (s, 3H, CH_3_). ^13^C-NMR (DMSO-*d*_6_): δ 157.0 (C_2_), 153.6 (C_12_), 148.8 (C_q_), 133.2 (C_9_), 132.6 (C_q_), 124.5 (C_3_), 124.2 (C_8_), 120.8 (C_4_), 116.4 (C_q_), 112.9 (C_q_), 103.1 (C_1_), 56.5 (OCH_3_), 35.2 (C_10_), 34.3 (C_6_), 28.0 (C_13_), 26.5 (C_7_), 25.6 (C_11_), 23.3 (CH_3_). HESI-HRMS: *m*/*z*: calcd. for C_18_H_21_N_2_O: 281.1609 [M + H]^+^ found 281.1623.

### 4.2. Biochemical Studies

#### 4.2.1. In Vitro Anti-Cholinesterase Assay

The inhibitory activities of (±)-**1**·HCl and all the standards against human recombinant AChE (*h*AChE, E.C. 3.1.1.7, purchased from Sigma-Aldrich, Prague, Czech Republic) and human plasmatic BChE (*h*BChE, E.C. 3.1.1.8, purchased from Sigma-Aldrich, Prague, Czech Republic) were determined using modified Ellman’s method [34,35,36] and are expressed as IC_50_ (the concentration of the compound that is required to reduce 50% of cholinesterase activity). Other used compounds were phosphate buffer solution (PBS, pH = 7.4), 5,5′-dithio-bis(2-nitrobenzoic) acid (Ellman’s reagent, DTNB), acetylthiocholine (ATCh), and butyrylthiocholine (BTCh) were also commercially available and were purchased from Sigma-Aldrich (Prague, Czech Republic). During the measurement, 96-well microplates from polystyrene (ThermoFisher Scientific, Waltham, MA, USA) were used. The solutions of corresponding enzyme in PBS were prepared up to final activity 0.002 U/µL. The assay medium (100 µL) consisted of cholinesterase (10 µL), DTNB (20 µL of 0.01 M solution) and PBS (40 µL of 0.1 M solution). The solutions of the tested compounds (10 µL of different concentrations) were pre-incubated for 5 min in the assay medium and then solution of substrate (20 µL of 0.01 M ATCh or BTCh iodide solution) was added to initiate the reaction. The increase of absorbance was measured at 412 nm using Multimode microplate reader Synergy 2 (BioTek Inc., Winooski, VT, USA). For the calculation of the resulting measured activity (the percentage of inhibition I) following formula was used:

(1) $$I=\left(1-\frac{\Delta A_{i}}{\Delta A_{0}}\right)\times 100$$

where Δ*A_i_* indicates absorbance change provided by adequate enzyme exposed to corresponding inhibitor and Δ*A*_0_ indicates absorbance change when a solution of PBS was added instead of a solution of inhibitor. Software Microsoft Excel (Redmont, WA, USA) and GraphPad Prism version 5.02 for Windows (GraphPad Software, San Diego, CA, USA) were used for the statistical data evaluation.

#### 4.2.2. Kinetic Study of AChE Inhibition

The kinetic study of *h*AChE was performed by using above mentioned Ellman’s method [34,35]. The concentrations of (±)-1·HCl used for the measurements were as follows: 1.00, 10.0, and 100 µM. The values of *V*_max_ and *K*_m_ of the Michaelis–Menten kinetics as well as the values of *K*_i_ and *K*_i’_ were calculated by non-linear regression from the substrate velocity curves. Linear regression was used for calculation of Lineweaver–Burk plots. All calculations were performed using GraphPad Prism software version 5.02 for Windows (San Diego, CA, USA).

#### 4.2.3. Determination of In Vitro Blood-Brain Barrier Permeation

For the prediction of (±)-**1**·HCl and (±)-HupY·HCl to passively penetrate the BBB, the PAMPA protocol was used [50,51]. Briefly, the filter membrane of the donor plate was coated with PBL (Polar Brain Lipid, Avanti, St. Normal, IL, USA) in dodecane (4 µL of 20 mg/mL PBL in dodecane) and the acceptor well was filled with 300 µL of PBS pH 7.4 buffer (VD). Tested compounds were dissolved first in DMSO and diluted with PBS pH 7.4 to reach the final concentration 100 µM in the donor well. Concentration of DMSO did not exceed 0.5% (*v*/*v*) in the donor solution. Next, 300 µL of the donor solution was added to the donor wells (VA) and the donor filter plate was carefully put on the acceptor plate so that coated membrane was “in touch” with both donor solution and acceptor buffer. Test compound diffused from the donor well through the lipid membrane (Area = 0.28 cm^2^) to the acceptor well. The concentration of the drug in both donor and the acceptor wells was assessed after 3, 4, 5, and 6 h of incubation in quadruplicate using the UV plate reader Synergy HT (Biotek, Winooski, VT, USA) at the maximum absorption wavelength of each compound. Concentration of the compounds was calculated from the standard curve and expressed as the permeability (Pe) according the equation [65,66]:

(2) $$log\;P_{e}=log\left\{C\times ln\left(1-\frac{{\left[drug\right]}_{aceptor}}{{\left[drug\right]}_{equilibrium}}\right)\right\}$$

where $C=\frac{\left(V_{D}\times V_{A}\right)}{\left(V_{D}+V_{A}\right)Area\times time}$.

#### 4.2.4. Evaluation of Cytotoxicity by MTT Assay

Cytotoxicity of tested compounds was evaluated by standard MTT assay [56] using the HepG2 cell line originally from human liver hepatocellular carcinoma, ACHN cell line of human renal adenocarcinoma, SH-SY5Y cell line of human neuroblastoma (ACTCC, USA). HepG2, ACHN, and SH-SY5Y cells were plated at density 17–30 × 10^3^ per well in a 96-well plate in Dulbecco’s Modified Eagle’s Medium (DMEM, Biowest, Riverside, MO, USA) or DMEM-F12 (Biowest, Riverside, MO, USA) with 10% FBS (Gibco, Langley, OK, USA) and were left unattached overnight. The incubation was performed under these conditions: 37 °C, 5% CO_2_ and 80–95% air humidity. Stock solutions of tested compounds were prepared in dimethyl sulfoxide (DMSO, Sigma-Aldrich, Prague, Czech Republic) and then diluted in DMEM or DMEM-F12 medium. The final concentration of DMSO was less than 0.5%.

The cell viability was detected using (3-4,5-dimethylthiazol-2-yl)-2,5-diphenyltetrazolium bromide (MTT; Sigma-Aldrich, Prague, Czech Republic) assay [56] after 24 h of incubation with compounds. Subsequently, the medium was aspirated and 10–100 µL MTT solution in serum- and phenol red-free DMEM medium was added to each well. Cells were then incubated for 1–3 h. The medium was then discarded and purple crystals of MTT formazan were dissolved in 100 μL DMSO under shaking. The absorbance was measured with a microplate (Biotek Inc., Winooski, VT, USA) at a test wavelength of 570 nm.

The IC_50_ values were calculated using four parametric nonlinear regression with statistic software GraphPad Prism (GraphPad Software, Inc., San Diego, CA, USA). Data were obtained from three independent experiments performed in duplicates or triplicates. IC_50_ values were expressed as mean ± SEM.

### 4.3. Molecular Modeling Studies

Two structures of *h*AChE and *h*BChE were gained from the RCSB Protein Data Bank–PDB ID: 4EY7 (crystal structure of *h*AChE) and 4BDS (crystal structure of *h*BChE) [63,64]. All receptor structures were prepared by the DockPrep function of UCSF Chimera (version 1.4) and converted to pdbqt-files by AutodockTools (v. 1.5.6) [67,68]. Flexible residues selection was based on previous experience (*h*AChE) or spherical region around the binding cavity [69,70,71,72,73,74,75].

Three-dimensional structures of ligands were built by Open Babel (v. 2.3.1), minimized by Avogadro (v 1.1.0), and converted to pdbqt-file format by AutodockTools [76]. The docking calculations were done by Autodock Vina (v. 1.1.2) with the exhaustiveness of 8 [77]. Calculation was repeated 15 times for each ligand and receptor and the best-scored result was selected for manual inspection. The visualization of enzyme–ligand interactions was prepared using 1.3. (The PyMOL Molecular Graphics System, Version 1.5.0.4 Schrödinger, LLC, Mannheim, Germany). 2D diagrams were created with PoseView software.

## 5. Conclusions

Although THA was withdrawn from the market due to its side effects, it still represents a valuable scaffold for the design of novel potential anti-AD agents. Examples of such compounds are 7-MEOTA and HupY. 7-MEOTA is a derivative of THA that has interesting pharmacological profile while having suppressed hepatotoxicity. HupY is a combination of THA and (–)-huperzine A, both being potent inhibitors of ChE, and as such showing high inhibitory activity against AChE. We report here the design, synthesis, and pharmacological profile of novel huprine, namely 2-methoxyhuprine, sharing structural features of 7-MEOTA and HupY. The design strategy of (±)-**1** yielded non-selective mixed type (on *h*AChE) ChEI with balanced physicochemical properties. In silico studies predicted that the more active enantiomer was 7*S*,11*S*-**1** for *h*AChE, while *h*BChE can be more potently inhibited by 7*R*,11*R*-**1**. The final confirmation of the work presented here requires firstly enantiomer separation followed by a biological assessment. Taken together, our study enriches the pipeline of lead compounds for further development of huprine derivatives potentially applicable for AD treatment.

## Figures and Tables

**Table 1:** Inhibitory activities of novel (±)-**1**·HCl and reference compounds ((±)-HupY·HCl, THA, 7-MEOTA and 6-chlorotacrine) for *h*AChE and *h*BChE expressed as IC_50_ values.

Compound	IC_50_ ± SEM (µM) ^a^		Selectiviy for *h*AChE ^b^
	*h*AChE	*h*BChE	
(±)-**1**·HCl	2.63 ± 0.36	3.76 ± 0.12	1.43
(±)-HupY·HCl	0.00164 ± 0.00014	0.358 ± 0.025	218.29
THA	0.32 ± 0.013 ^c^	0.08 ± 0.001 ^c^	0.68
7-MEOTA	10.00 ± 0.974 ^c^	17.56 ± 0.795 ^c^	1.76
6-chlorotacrine	0.02 ± 0.001 ^c^	1.78 ± 0.097 ^c^	100.68

^a^ Results are expressed as the mean of at least three experiments; ^b^ selectivity for *h*AChE is determined as ratio IC_50_(*h*BChE)/IC_50_(*h*AChE); ^c^ data taken from Ref. [45,46,47,48].

**Table 2:** In vitro BBB permeability prediction for (±)-**1**·HCl and (±)-HupY·HCl as positive reference compound.

Compound	BBB Penetration Estimation	
	*P*_e_ ± SEM (10^−6^ cm·s^−1^) ^a^	CNS (+/−) ^b^
(±)-**1**·HCl	7.64 ± 0.22	CNS +
(±)-HupY·HCl	5.89 ± 0.36	CNS +
THA	5.30 ± 0.20 ^c^	CNS +
Donepezil	7.3 ± 0.9	CNS +
7-MEOTA	6.50 ± 1.85	CNS +
6-chlorotacrine	5.00 ± 0.45 ^c^	CNS +
Cefuroxim	2.70 ± 0.1	CNS −
Piroxicam	2.20 ± 0.15	CNS −

^a^ Results are expressed as the mean of at least three experiments; ^b^ CNS +: high BBB permeation predicted with *P*_e_ (10^−6^ cm·s^−1^) > 4.0, CNS −: low BBB permeation predicted with *P*_e_ (10^−6^ cm·s^−1^) < 2.0, CNS +/−: BBB permeation uncertain with *P*_e_ (10^−6^ cm·s^−1^) from 4.0 to 2.0; ^c^ taken from references [52,53].

**Table 3:** Psychicochemical properties calculated by MarvinSketch 15.1.19.0 (Budapest, Hungary) and CNS MPO scores for (±)-**1**·HCl and standards under the survey.

Compound	Physicochemical Properties						CNS MPO Score ^b^
	Clog*P* ^a^	Clog*D*_7.4_^a^	TPSA ^a^	MW ^a^	HBD ^a^	Cp*K*_a_^a^	
(±)-**1**·HCl	2.91 (3.58 ± 0.02) ^c^	1.56 (1.734) ^c^	49.39	280.36	2	8.91 (9.54 ± 0.02) ^c^	5.0
(±)-HupY·HCl	3.67	2.95	40.16	285.79	2	8.07	5.0
THA	2.63	1.25	40.16	199.27	2	8.95	4.7
7-MEOTA	2.47	1.08	49.39	229.30	2	8.98	5.0
6-chlorotacrine	3.23	2.41	40.16	233.72	2	8.20	5.1

^a^ The optimal values for each specific physiochemical attribute for CNS active compounds is postulated to range in the following intervals: Clog*P* = 1–5; Clog*D*_7.4_ = 0–3; TPSA ≤ 60–70; MW ≤ 400 Da; HBD ≤ 3; Cp*K*_a_ = 4–10; ^b^ optimal CNS MPO score = 4–6, calculated according to protocol from [54,55]; ^c^ experimentally determined values with Sirius T3 device.

**Table 4:** Results of cytotoxicity of (±)-**1**·HCl and reference compounds in HepG2, ACHN, and SH-SY5Y cell lines.

Compound	IC_50_ ± SEM (µM) ^a^		
	HepG2	ACHN	SH-SY5Y
(±)-**1**·HCl	14.91 ± 0.11	32.22 ± 0.72	17.84 ± 0.09
(±)-HupY·HCl	15.87 ± 1.17	24.62 ± 0.32	17.49 ± 0.44
THA	168.47 ± 3.63	155.20 ± 0.67	120.63 ± 1.97
7-MEOTA	44.37 ± 3.35	49.27 ± 2.69	26.83 ± 1.04
6-chlorotacrine	43.20 ± 1.17	55.11 ± 1.76	50.40 ± 1.28

^a^ Results are expressed as the mean of at least three experiments, cytotoxic profile assessed after 24 h.
